# A Value-Based Framework for CLL Care: Results of a Scoping Review

**DOI:** 10.3390/cancers18213462

**Published:** 2026-10-27

**Authors:** Stefano Molica, David Allsup

**Affiliations:** 1 Clinical Sciences Centre, Hull York Medical School, University of Hull, Hull HU16 5JQ, UK; 2 Centre for Biomedicine, Hull York Medical School, Hull HU16 5JQ, UK; david.allsup@hyms.ac.uk

**Keywords:** chronic lymphocytic leukemia (CLL), targeted inhibitors (or BTK/BCL-2 inhibitors), value-based framework, health-related quality of life (HRQoL), health-system stewardship (or resource allocation)

## Abstract

Background: Evaluating multidimensional value in chronic lymphocytic leukemia (CLL) is complicated by distinct endpoint hierarchies, patient heterogeneity, therapeutic resistance mechanisms, and healthcare costs. Consequently, addressing these multifaceted aspects of CLL management remains a significant challenge. This scoping review integrates comparative clinical evidence, surrogate endpoints, and health-economic data to formulate a value-based framework for personalized therapeutic decision-making. Methods: A literature search was conducted across PubMed/MEDLINE, Embase, the Cochrane Library, and ClinicalTrials.gov from inception through June 2026. Studies were independently screened and extracted by two reviewers (S.M. and D.A.). Methodological quality and risk of bias were evaluated narratively across phase III randomized controlled trials, real-world cohorts, and economic models. Results: This scoping review evaluated 65 core publications that met stringent eligibility criteria, supplemented by 22 secondary contextual references. Findings demonstrate that continuous covalent Bruton tyrosine kinase inhibitor (BTKi) therapy provides sustained disease control, with a median progression-free survival (PFS) of up to 8.9 years in the RESONATE-2 trial, albeit at the cost of persistent low-grade toxicities and long-term financial burden. Conversely, fixed-duration, venetoclax-based regimens induce deep and durable remissions (median PFS of 104.9 months in IGHV-mutated patients in the 9-year follow-up of the CLL14 trial). By enabling treatment-free intervals, fixed-duration strategies mitigate cumulative toxicity, lower long-term healthcare costs (including a 67% post-treatment cost reduction in real-world Medicare analyses), and improve health-related quality of life (HRQoL). Furthermore, methodological considerations—such as surrogate endpoints (e.g., undetectable measurable residual disease [uMRD] and time to next treatment [TTNT]), which remain susceptible to immortal time bias, alongside monitoring frequency—are paramount in the comparative assessment of CLL trials. In clinical practice, incorporating the ESMO Magnitude of Clinical Benefit Scale for Hematological Malignancies (ESMO-MCBS:H v1.0) supports structured, patient-centered selection of therapy. Conclusions: Deciphering CLL management requires synthesizing genomic risk profiles (*TP53* aberrancy, *IGHV* mutational status), patient physiological reserve, adverse event profiles, and resource utilization. We propose the GUIDE framework (Genetics, Unique host factors, Individual preferences, Delivery logistics, Expectations) as a conceptual model to operationalize value-based care in routine clinical practice.

## Simple Summary

The paradigm shift in chronic lymphocytic leukemia (CLL) management—from conventional chemoimmunotherapy to targeted small-molecule inhibitors of B-cell receptor and anti-apoptotic pathways—has exposed the limitations of traditional trial endpoints. Standard metrics such as progression-free survival (PFS) fail to adequately account for net clinical value, cumulative toxicity profiles, and long-term patient burden across multi-decadal treatment courses. Synthesizing evidence across PubMed, Embase, and ClinicalTrials.gov pursuant to PRISMA guidelines, this systematic analysis evaluates targeted agents (including covalent and non-covalent BTK inhibitors and BCL-2 inhibitors) through a value-based framework. The findings indicate that incremental therapeutic efficacy cannot be assessed in isolation; rather, it must be contextualized alongside health-related quality of life (HRQoL), adverse event profiles, treatment-free intervals, and health-system stewardship. Achieving optimal value-based care requires ongoing clinical calibration between biological precision, patient-centered utility, and sustainable resource allocation throughout the disease continuum.

## 1. Introduction

The history of chronic lymphocytic leukemia (CLL) therapy reflects a steady evolution in how hematologists define treatment success [1]. Conventional chemoimmunotherapy regimens, while capable of producing deep remissions in select favorable-risk subsets of affected patients, were inherently constrained by myelosuppression, infection risk, organ toxicity, and secondary malignancies [2]. The advent of targeted small-molecule inhibitors of the B-cell receptor signaling and anti-apoptotic pathways has fundamentally modified the therapeutic landscape of CLL [3].

Because CLL remains largely incurable and predominantly affects older populations, the management of this disease is defined not by a single intervention, but by a multi-decadal treatment and management approach [4]. Although historically characterized as an indolent leukemia, the lived experience of persons with CLL involves prolonged medical surveillance, persistent disease-related fatigue, immunocompromise, and the underlying psychological weight of an incurable diagnosis [5]. Consequently, the overarching treatment aims have evolved beyond the establishment of superior efficacies of novel regimens to the determination of the net clinical value of such interventions [6]. The achievement of this aim will require a comprehensive evaluation that weighs the incremental therapeutic gains associated with novel, targeted therapies against the associated toxicity profiles, exposure duration, and cumulative treatment burden [7].

Current clinical trial designs, whilst optimized for quantitative endpoints like progression-free survival (PFS), remain only partially suited to evaluate these multifaceted patient-centered metrics [1]. Reinterpreting trial evidence through a value-based framework—a model that systematically balances clinical efficacy against overall health system costs and burden—demands a broader selection of therapeutic endpoints. Rather than relying solely on traditional survival outcomes, this perspective incorporates health-related quality of life (HRQoL), drug toxicity profiles, treatment-free duration, and prudent long-term resource management [1,8].

In this scoping review, we analyze how value-based frameworks can be applied to contemporary CLL therapies to better capture their true clinical utility. Specifically, we evaluate clinical trial evidence to determine the magnitude of clinical benefit across key domains, including HRQoL, adverse event burdens, treatment-free intervals, and long-term healthcare stewardship.

## 2. Literature Search Strategy and Selection

A scoping review was conducted in accordance with the Preferred Reporting Items for Systematic Reviews and Meta-Analyses extension for Scoping Reviews (PRISMA-ScR) guidelines and has not been registered (Supplementary Table S1). The primary objective was to map the broad evidence base regarding targeted therapies in CL [9]. A systematic literature search was executed across PubMed/MEDLINE, Embase (via Elsevier), and ClinicalTrials.gov/Cochrane Library to identify relevant studies published up to June 2026. The full search strategies, including exact keywords, are provided in Supplementary Material. To minimize selection and reporting bias, title/abstract screening and full-text eligibility assessments were performed independently by two reviewers ([S.M.] and [D.A.]). Any discrepancies or methodological incongruences regarding study inclusion, risk of bias, or data extraction were systematically identified and resolved through iterative consensus discussions. In accordance with PRISMA-ScR standards, formal critical appraisal or risk-of-bias scoring of individual studies were not conducted, as the primary goal of this scoping review was evidence mapping rather than meta-analytic effect synthesis; potential sources of bias across included studies (e.g., publication bias, industry sponsorship, and retrospective real-world study designs) were instead evaluated and discussed narratively.

**Figure 1:**
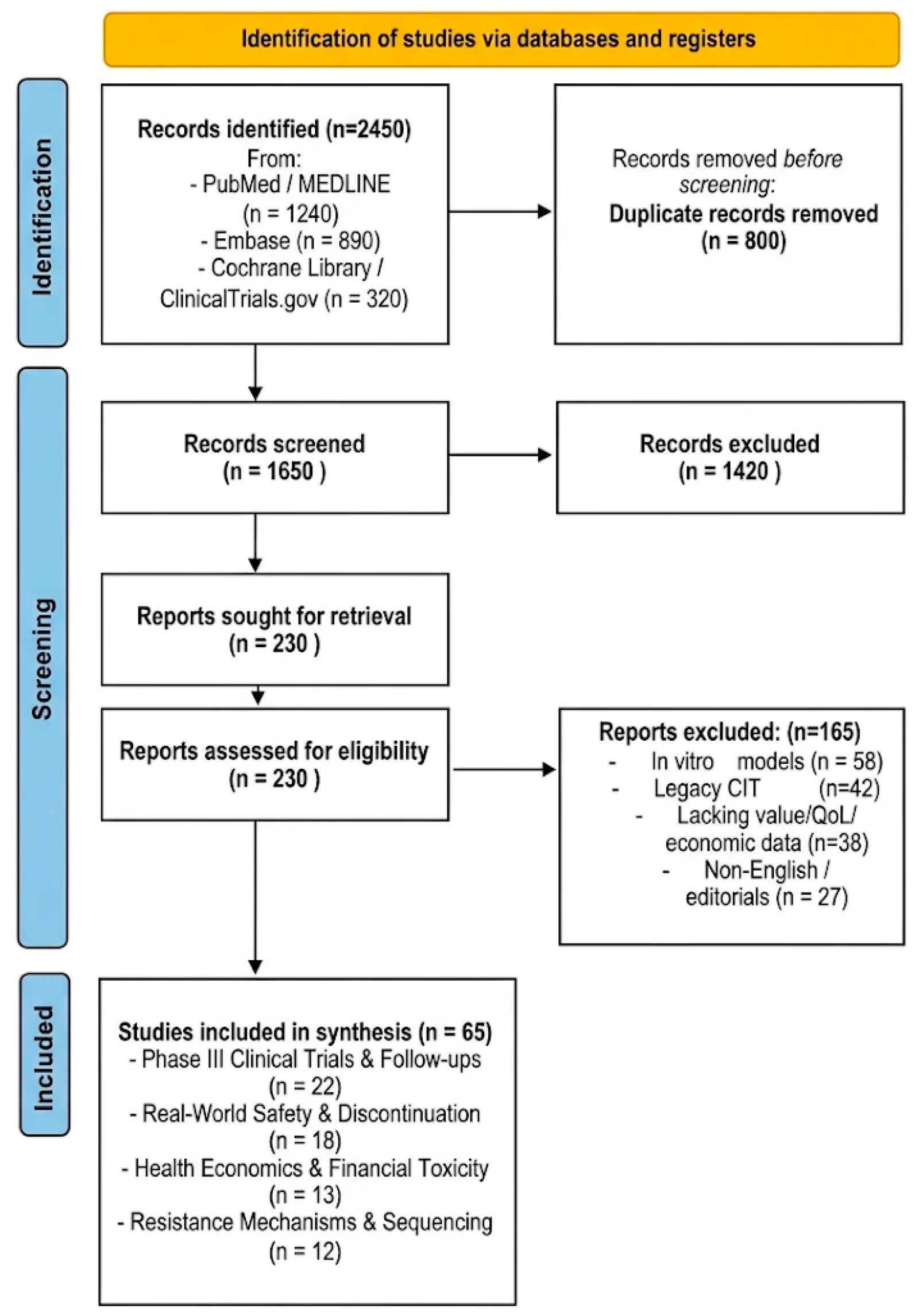
PRISMA flow diagram of literature identification, screening, and inclusion.

The search yielded an initial total of 2450 records (PubMed/MEDLINE: n = 1240; Embase: n = 890; Cochrane Library/ClinicalTrials.gov: n = 320). Following the removal of 800 duplicate records, 1650 unique titles and abstracts were screened, resulting in the exclusion of 1420 non-relevant citations. A total of 230 full-text reports were sought for retrieval and assessed for eligibility. Of these, 165 reports were excluded based on predefined criteria: utilization of pre-clinical or in vitro models (n = 58), focus on legacy chemoimmunotherapy (CIT) regimens lacking modern targeted comparator arms (n = 42), lack of relevant clinical value, quality-of-life (QoL), or health economic endpoints (n = 38), and non-English text or editorial commentaries (n = 27). Ultimately, 65 core publications fulfilled all scoping requirements and were selected for evidence mapping and narrative synthesis (Figure 1), categorized into Phase III clinical trials and follow-ups (n = 22), real-world safety and discontinuation (n = 18), health economics and financial toxicity (n = 13), and resistance mechanisms and sequencing (n = 12) [9]. Additionally, 22 supplementary references were included to provide complementary context and broader perspective to this scoping review.

## 3. Continuous Versus Fixed-Duration Paradigms: Defining Clinical and Therapeutic Value

Contemporary CLL care is defined by two distinct treatment paradigms: continuous targeted therapy and fixed-duration regimens [5]. Each imposes a fundamentally different timeline of disease management and cumulative treatment burden on both patients and healthcare systems [5]. Notably, comparing continuous treatment paradigms with fixed-duration regimens introduces structural statistical challenges, notably, immortal time bias and guarantee-time bias. Immortal time bias occurs when patients must survive or remain progression-free throughout an initial fixed-duration treatment phase or until achieving a designated landmark (e.g., undetectable measurable residual disease [uMRD] at month 12 or 15) to be included in off-treatment outcome cohorts. This creates a period during which the event of interest cannot occur, artificially inflating the apparent efficacy of the fixed-duration arm. Similarly, guarantee-time bias can distort comparisons if early progressors or patients discontinuing therapy during the active phase are excluded from landmark analyses of treatment-free survival. Rigorous comparative evaluations require prospective landmark designs, intention-to-treat protocols, and statistical adjustments (such as time-dependent covariates) to account for these temporal artifacts.

### 3.1. Continuous Targeted Therapy: Open-Ended Suppression

Continuous covalent Bruton tyrosine kinase (cBTK) inhibitor monotherapy functions as a long-term disease-suppression strategy. Rather than eradicating the leukemic clone within a defined treatment window, administration is maintained indefinitely until disease progression or unacceptable toxicity occurs [10]. This paradigm boasts the longest mature follow-up data among targeted agents CLL. Notably, 10-year follow-up from the RESONATE-2 trial demonstrated a median PFS of 8.9 years with frontline ibrutinib, maintaining durable disease control across high-risk genomic subsets, including patients with unmutated *IGHV*, del(11q), or *TP53* alterations [11].

Building upon these outcomes, second-generation cBTK inhibitors have demonstrated comparable or superior efficacy coupled with favorable tolerability profiles. In the ELEVATE-TN trial, 6-year follow-up of acalabrutinib confirmed sustained remissions, yielding at 72-month follow-up PFS rates of 78% in combination with obinutuzumab and 61.5% as monotherapy [12]. Similarly, 6-year results from the SEQUOIA trial underscore the long-term durability of zanubrutinib, which achieved a 74% 6-year PFS rate and a 72% reduction in the risk of progression or death compared to CIT in patients without del(17p) [13].

To address the cumulative off-target toxicities and dose interruptions associated with first-generation therapy, second-generation cBTK inhibitors were evaluated against ibrutinib in pivotal head-to-head trials. In the Phase 3 ELEVATE-RR trial in previously treated high-risk CLL, acalabrutinib demonstrated non-inferior PFS compared to ibrutinib (median 38.4 months in both arms), while significantly reducing the incidence of cardiovascular toxicity—most notably lower rates of atrial fibrillation/flutter (9.4% vs. 16.0%)—and yielding fewer adverse-event-related discontinuations [14]. Advancing this class further, the randomized ALPINE trial in relapsed/refractory (R/R) CLL established that zanubrutinib achieved statistical superiority in PFS over ibrutinib (HR 0.68) alongside higher overall response rates [15]. Importantly, zanubrutinib exhibited a superior cardiovascular safety profile over ibrutinib, marked by a lower incidence of atrial fibrillation/flutter (7.1% vs. 17.0%) and zero cardiac deaths compared with six in the ibrutinib arm [15].

The clinical convenience of continuous therapy, however, must be balanced against the cumulative burden of indefinite treatment. Each additional year of disease control entails continued exposure to therapy and, consequently, prolonged risks of persistent toxicities—including gastrointestinal symptoms, arthralgias, hypertension, bleeding, and fatigue—that may progressively impair functional capacity [12,16]. Continuous oral therapy may also impose a less tangible psychological burden, as the daily need to take antineoplastic medication can serve as a persistent reminder of living with an active malignancy and reinforce a sense of ongoing illness. From a healthcare-system perspective, indefinite treatment requires continued clinic visits, laboratory monitoring, and sustained pharmaceutical expenditure [17,18,19]. Finally, prolonged therapeutic selective pressure may favor the emergence of resistant disease, including the acquisition of *BTK* and *PLCG2* alterations, potentially compromising subsequent treatment options [20,21].

The therapeutic paradigm of continuous BTK inhibition is expanding with the development of non-covalent BTK inhibitors, including pirtobrutinib [22]. Data from the randomized Phase III BRUIN-CLL-313 and BRUIN-CLL-314 trials demonstrate that pirtobrutinib achieves superiority over CIT in treatment-naïve (TN) patients and non-inferiority to ibrutinib in both TN cohorts and patients with R/R disease lacking prior covalent BTK inhibitor exposure [23,24]. Although these findings do not warrant an immediate alteration in clinical practice, they provide a compelling rationale for transitioning pirtobrutinib from late-line R/R CLL to earlier treatment settings [23,24]. Importantly, despite overcoming the resistance-conferring C481S mutation, pirtobrutinib therapy may still select for secondary gatekeeper and kinase-dead mutations during disease progression [25].

To overcome emerging resistance mechanisms associated with continuous covalent and non-covalent BTK inhibitors (such as *BTK^C^*^481*S*^ or kinase-dead mutations like *BTK^L^*^528*W*^) as well as BCL-2 mutations (e.g., *BCL*2*^Gly^*^101*Val*^), novel therapeutic modalities are rapidly expanding the CLL armamentarium [26]. Beyond non-covalent BTKi inhibition, targeted protein degradation—principally through BTK Proteolysis Targeting Chimeras (PROTACs) such as BGB-16673, NX-2127, and NX-5948—represents a transformative strategy [26]. Notably, the discussion of BTK/BCL-2 inhibitor resistance should be expanded to include protein degradation and stress-response approaches [27].

Unlike traditional small-molecule enzymatic inhibitors, BTK degraders recruit E3 ubiquitin ligases to induce selective ubiquitin-proteasomal degradation of both wild-type and mutant BTK proteins, thereby abolishing scaffold-dependent signaling even in the setting of kinase-dead or gatekeeper resistance. In parallel, targeting cellular stress-response pathways is emerging as a compelling approach to circumvent resistance [26]. Modulating heat shock proteins, the unfolded protein response (UPR), endoplasmic reticulum stress pathways, and downstream microenvironmental survival cues provides synergistic opportunities when combined with BTK or BCL-2 targeted agents. Integrating targeted protein degraders and stress-response modulators into combination and sequencing strategies holds significant promise for restoring therapeutic sensitivity and sustaining long-term disease control in double- or triple-refractory CLL cohorts [26].

### 3.2. Fixed-Duration Targeted Therapy: Temporal Bounding and Off-Treatment Liberation

Fixed-duration regimens operate on the core principle of achieving deep responses—such as uMRD—within a specified timeframe, thereby enabling planned treatment discontinuation [28]. Venetoclax-based combinations—administered for 12 months in frontline treatment or 24 months in R/R settings—demonstrate that achieving uMRD leads to durable, multi-year treatment-free remissions [29,30,31,32,33,34,35].

Data presented at the 2026 European Hematology Association (EHA) Congress reinforce fixed-duration therapy as an emerging standard of care. Long-term follow-up from landmark trials evaluating venetoclax plus obinutuzumab—most notably the 9-year update of the CLL14 trial (representing the longest follow-up for frontline fixed-duration regimens to date) alongside the GAIA/CLL13 trial—collectively position uMRD as an important surrogate trial endpoint [35,36].

Across pivotal trials evaluating fixed-duration venetoclax–BTK inhibitor combinations (e.g., CAPTIVATE, GLOW, AMPLIFY, and CLL17), long-term efficacy remains consistent despite substantial baseline heterogeneity in cohort size, median age, fitness, comorbidity burden, and high-risk genomic features [29,30,34,35]. Notably, findings from the CLL17 trial—which evaluated fixed-duration regimens (ibrutinib plus venetoclax [IV] or venetoclax plus obinutuzumab [VO]) against continuous ibrutinib in an all-comers, previously untreated CLL population—demonstrated non-inferiority for both fixed-duration regimens in comparison to continuous ibrutinib regarding investigator-assessed PFS [35].

The primary clinical trade-off associated with fixed-duration therapy lies in its initial logistical and management complexity. Venetoclax initiation mandates a structured dose-escalation schedule, rigorous risk stratification for tumor lysis syndrome (TLS), potential short-term hospitalization for high-risk patients, frequent laboratory monitoring, and intravenous infusions when administered with anti-CD20 monoclonal antibodies [32,37]. Furthermore, acute toxicities—predominantly transient neutropenia, infectious complications, and infusion-related reactions—are similarly clustered within the initial months of treatment [32,33].

However, this operational and clinical burden is inherently time-limited. Upon completion of treatment, patients enter an off-treatment surveillance phase in which they are no longer exposed to daily antineoplastic therapy and its associated toxicities. This treatment-free interval (TFI) reduces the burden of chronic drug-related adverse events, frequent clinical visits, and ongoing out-of-pocket expenses, while potentially providing patients with greater freedom from treatment-related constraints [29,30,31,32,33,34,35,36]. These benefits may translate into meaningful improvements in HRQoL that extend beyond conventional efficacy and safety endpoints [29,30,31,32,33,34,35,36].

A core theoretical advantage of fixed-duration venetoclax-based therapy is the option for a drug holiday and subsequent retreatment upon disease progression [38]. However, establishing the true net value of fixed-duration therapy requires longer-term comparative clinical data evaluating the efficacy, duration of second response, and overall cost-effectiveness of retreatment versus switching to an alternative therapeutic class.

Real-world evidence consistently demonstrates quality-of-life (QoL) improvements following the completion of venetoclax-based fixed-duration therapy. In the observational PROVIDENCE study, patients receiving fixed-duration regimens achieved gains in global health status, physical and role functioning, symptom burden, pain, and fatigue; conversely, those on continuous therapy experienced a significant decline in global QoL alongside increased symptom burden [39]. Similarly, in older, unfit populations, fixed-duration VO improved global health status, physical and emotional functioning, fatigue, and dyspnea while mitigating geriatric impairments during treatment [40]. Prospective data from the real-world VeRVe study further demonstrate global QoL gains that persisted and continued to improve after treatment cessation [41]. Collectively, these real-world findings validate the therapeutic value of first-line fixed-duration VO and complement phase 3 trial QoL reports from CLL14 and GAIA/CLL13 [42,43].

While research on fixed-duration combination regimens has largely focused on first-line treatment, data regarding R/R CLL remain sparse [31]. The Phase 3 BRUIN CLL-322 trial evaluated whether adding the noncovalent BTK inhibitor pirtobrutinib to fixed-duration venetoclax-rituximab (VRP) improves outcomes compared to VR alone in previously treated CLL [44]. The trial met its primary endpoint, demonstrating a statistically significant improvement in PFS by independent review committee assessment. Adding pirtobrutinib to VR reduced the risk of disease progression or death by 45.3% compared to VR monotherapy. Importantly, BRUIN CLL-322 enrolled a contemporary cohort reflective of current clinical practice—including a high proportion of patients previously treated with cBTKi therapy—establishing vital Phase 3 benchmark data for VR in this increasingly prevalent population [29,44].

## 4. Value-Based Therapeutic Selection

Value-based therapeutic selection in CLL requires aligning a regimen’s specific burden profile with both the biological disease risk and the patient’s individual clinical context.

### 4.1. Biological and Clinical Variables

The 2026 EHA CLL guidelines highlight genomic markers—principally *TP53* aberrations and *IGHV* mutational status—as central to prognostic risk stratification [45]. For *TP53*-aberrant disease, continuous BTK inhibition remains the preferred standard of care, with second-generation covalent inhibitors (acalabrutinib, zanubrutinib) favored over ibrutinib due to superior selectivity and lower cardiovascular toxicity [45].

Although venetoclax-based doublets and triplets achieve high uMRD rates in *TP53*-aberrant CLL, limited long-term evidence precludes routine fixed-duration use outside selected cases pending mature post-discontinuation durability data [30,35,46,47]. For non-high-risk profiles (*TP53* wild-type), fixed-duration frontline therapy is supported by robust long-term clinical trial data [27,28,29,30,31,32,33]. Specifically, 110-month median follow-up from the CLL14 trial demonstrated a median PFS of 104.9 months with fixed-duration VO in *IGHV*-mutated disease [36]. Furthermore, the final 5.5-year analysis of the CAPTIVATE cohort showed that fixed-duration ibrutinib–venetoclax yields deep, durable responses in unmutated *IGHV* disease, achieving a 5.5-year PFS rate of 55% [48].

Tailoring these regimens requires evaluating each patient’s individual physiological reserve [1]. Because the median age at diagnosis is 72 years and octogenarians constitute 20% of real-world cohorts, clinical trial data from younger, fitter populations inherently underestimate real-world toxicities and discontinuation rates [49,50]. Addressing this gap necessitates evaluating “biological age”—a dynamic measure of physiological reserve that deteriorates under leukemic or infectious stress but may partially recover following remission—to guide personalized treatment selection [51,52]. While formal Comprehensive Geriatric Assessment (CGA) remains the gold standard for assessing multidimensional fitness, its resource-intensive nature limits routine outpatient implementation [52]. Consequently, pragmatic screening tools—such as the Clinical Frailty Scale (CFS), Geriatric 8 (G8), and Fried Frailty Phenotype—effectively capture patient vulnerability to predict Grade 3–4 toxicities, PFS, and treatment discontinuations [53,54,55,56,57], although their routine clinical integration remains limited [50]. To translate these concepts into practice, recent EHA recommendations incorporate decision-support “heat maps” that convert risk and fitness metrics into actionable treatment strategies [45].

### 4.2. Value of Personalized Dosing Modifications

Recent evidence supports personalized dosing modifications over upfront treatment discontinuation [58]. In continuous BTKi therapy, post-hoc and prospective analyses (e.g., the TAILOR trial) demonstrate that proactive dose reductions (e.g., reducing ibrutinib from 420 mg to 280 mg daily) significantly reduce Grade 3+ toxicities while maintaining target receptor occupancy, disease control, and PFS [58].

In the fixed-duration cohort of the prospective Phase 2 TAILOR study, investigators evaluated reactive versus proactive ibrutinib dose adjustments (DA) in combination with venetoclax (I + V) for previously untreated CLL (*N* = 171) [59]. Following a 3-cycle ibrutinib lead-in, patients received either reactive DA per updated prescribing guidelines (Cohort 1A) or proactive ibrutinib reduction to 280 mg QD (Cohort 1B). Both cohorts met the primary endpoint, demonstrating statistically significant best overall response rates (94.2% [1A] vs. 89.4% [1B]; *p* < 0.05 vs. >75% historical benchmark). At a 14.6-month median follow-up, 12-month PFS and OS exceeded 97% across both arms. Proactive DA substantially reduced Grade 3 neutropenia (15.3% vs. 30.6%) while maintaining robust first-line efficacy [59].

To balance efficacy with cumulative toxicity in vulnerable populations, intermittent therapeutic strategies are under investigation. The randomized phase 2 STAIR trial evaluated fixed 18-month acalabrutinib monotherapy versus continuous acalabrutinib in patients with a median age of 77 years. Although continuous therapy maintained a superior 1-year PFS (96.3% vs. 53.1%), patients in the fixed-duration arm who relapsed achieved an 87% objective response rate upon drug re-challenge [60]. These findings, along with ongoing studies like the randomized STATIC trial, support the clinical feasibility of intermittent, treatment-limited schedules for frail patients [61].

## 5. The Value of Active Toxicity Stewardship

Traditional clinical trial reporting appropriately emphasizes grade 3–4 adverse events (AEs), serious AEs, treatment discontinuation, and treatment-related mortality [50,51]. Although essential, these metrics often underestimate the overall burden of long-term targeted therapies [50]. For patients receiving BTKi continuous therapy over extended periods, low-grade (grade 1–2) persistent AEs—such as fatigue, diarrhea, arthralgia, hypertension, bruising, or musculoskeletal symptoms—can severely impair daily physical activity, sleep, workplace productivity, and social functioning, thereby exerting a greater cumulative impact than transient severe events [11,12,13,14,15,16].

O’Connell et al. [62] evaluated data from 510 patients in the phase III ECOG-ACRIN E1912 trial comparing ibrutinib plus rituximab (IR) to fludarabine, cyclophosphamide, and rituximab (FCR). Using Bayesian mixed models, the investigators analyzed the impact of per-cycle counts of Grade 1 and Grade 2 AEs on two primary endpoints: patient side-effect bother—measured via the FACT-G GP5 item—and treatment discontinuation. Each additional Grade 1 AE per cycle was associated with a 13% increase in the odds of elevated side-effect bother (odds ratio [OR] = 1.13), whereas each Grade 2 AE increased the odds by 35% (OR = 1.35). Furthermore, each additional Grade 2 AE increased the odds of treatment discontinuation by 59% (OR = 1.59). The authors concluded that the omission of low- and moderate-grade toxicities from trial reporting obscures key determinants of patient burden and treatment cessation. Consequently, active monitoring of all-grade symptomatic AEs is critical for early identification of vulnerable patients, facilitating timely supportive care and enhancing therapeutic adherence [62].

Effective toxicity management relies on a systematic framework that goes beyond simple AE incidence to evaluate duration, resource utilization, quality-of-life impact, and mitigability through treatment adaptation [63]. First conceptualized in CLL clinical literature by Seymour et al. [64], this temporal paradigm shifts the focus of AE analysis from maximal toxicity grades to cumulative burden, persistence, and onset kinetics during prolonged drug exposure. Whereas conventional oncology trial design measures safety within standardized 28-day cycles, modern targeted CLL regimens—encompassing continuous BTK inhibitors and fixed-duration combinations—require dynamic, longitudinal models to fully characterize long-term tolerability.

## 6. Integrating Patient-Centered and Surrogate Endpoints to Define Therapeutic Value

When evaluating therapeutic value in CLL, it is vital to recognize that primary regulatory endpoints such as PFS, OS, patient-reported endpoints (e.g., HRQoL), and real-world or surrogate metrics (e.g., TTNT, MRD, and TFI) represent distinct clinical constructs and are not interchangeable. While regulatory endpoints quantify strict disease control or survival, patient-reported metrics capture subjective treatment burden, and surrogate biomarkers such as MRD reflect depth of response. Conflating these domains risks misinterpreting net clinical benefit.

### Real-World Endpoints: Time to Next Treatment and Treatment-Free Survival

TTNT serves as a pragmatic real-world clinical endpoint measuring the interval from initial therapy to the initiation of subsequent treatment [65,66,67]. Unlike PFS, TTNT evaluates overall net clinical benefit by integrating therapeutic efficacy, patient adherence, quality of life, tolerability, and physician decision-making [68]. However, regulatory authorities and health technology assessment bodies (e.g., FDA, EUnetHTA) caution against using TTNT as a direct surrogate endpoint despite strong trial-level correlations with PFS [69,70]. The metric remains vulnerable to methodological biases—such as variable follow-up durations, censoring patterns, and heterogeneous salvage therapies—as well as external non-biological factors including regional drug access, local resource constraints, and evolving standards of care [70,71]. For example, restricted drug availability may artificially prolong TTNT, whereas immediate access to novel agents may shorten it [70,71]. Consequently, formal regulatory adoption requires a two-level validation framework demonstrating patient- and trial-level concordance, alongside standardized retreatment criteria and individual patient data meta-analyses to systematically control for operational bias [70,71].

Complementing TTNT, Treatment-Free Survival (TFS) offers a vital metric for evaluating targeted regimens by quantifying the duration during which patients remain alive without active therapy, regardless of persistent or measurable residual disease [72].

In CLL, regimens yielding comparable PFS can produce vastly different patient experiences: continuous-dosing regimens demand indefinite daily treatment, whereas fixed-duration strategies typically deliver 12 to 24 months of therapy followed by prolonged treatment-free intervals [71]. Off-treatment periods spare patients cumulative drug toxicities, daily adherence burdens, frequent clinical monitoring, and ongoing financial expenditures while providing psychological relief from continuous medicalization [71]. Patient-preference studies further confirm that individuals frequently accept higher initial toxicity or complexity in exchange for fixed-duration treatment and sustained observation [73]—highlighting a critical limitation of traditional PFS hazard ratios in capturing patient-centered clinical value.

## 7. Measurable Residual Disease: As a Surrogate Endpoint

MRD has become one of the most informative indicators of response depth in CLL [71]. uMRD is consistently associated with improved disease control following fixed-duration treatment [27,28,29,30,31,32,33]. This has generated considerable interest in using MRD not only as a prognostic biomarker but also as a potential tool for treatment personalization [74].

This approach is supported in MRD-guided therapy. The FLAIR clinical trial experience represents an important example of response-adapted treatment duration. In this innovative trial, MRD-directed ibrutinib–venetoclax improved progression-free survival as compared to either FCR or continuous ibrutinib [75]. Notably, such strategies move treatment duration away from a uniform calendar-based model toward a biologically informed approach [74].

However, MRD assays vary in sensitivity, specimen type, methodology, and timing [76]. Crucially, the relationship between MRD status and long-term outcomes depends on the specific treatment regimen. Furthermore, effective subsequent therapies can attenuate the correlation between early surrogate endpoints (such as MRD negativity) and OS. Consequently, achieving MRD negativity should not automatically be interpreted as a universal indicator that treatment can safely be discontinued [76].

The appropriate future direction of MRD as a measure of patient outcomes is therefore of prospective validation. Trials should define the assay, threshold, timing, and stopping rule in advance and should evaluate whether MRD-guided treatment actually improves the balance between disease control and treatment burden [77].

Although response-adapted, MRD-guided treatment algorithms represent an attractive model for toxicity and financial stewardship, operationalizing this approach globally requires overcoming significant logistical barriers, as standardized high-sensitivity next-generation sequencing (NGS) and multi-color flow cytometry assays remain unequally distributed and variably reimbursed outside academic centers [74].

The pan-European phase III CLL18/MOIRAI trial is investigating whether individualization of treatment duration according to depth of response can improve outcomes beyond those achieved with a predefined fixed-duration regimen in treatment-naïve patients with CLL [78].

## 8. Integrating Clinical Benefit Scales and Health Technology Assessments in CLL Care

Determining true therapeutic value in CLL requires an examination beyond relative statistical significance toward objective frameworks that quantify meaningful clinical impact, long-term safety, and health-system sustainability [1]. To address this challenge, the European Society for Medical Oncology established the Magnitude of Clinical Benefit Scale for Hematological Malignancies (ESMO-MCBS:H v1.0), which grades treatment regimens. Under the ESMO-MCBS:H framework, therapies are scored on a descending scale where Grades 4 and 5 represent substantial clinical value, while Grades 1 through 3 reflect intermediate or marginal benefit [79]. Notably, the European Hematology Association (EHA) integrated this framework directly into its practice guidelines to prioritize regimens that deliver substantial clinical benefit [45].

To ensure rigorous evaluation and comparability across studies, we reviewed clinical benefit graded in accordance with ESMO-MCBS:H v1.0 guidelines, which was evaluated across five primary domains: survival outcomes (OS, PFS, DFS, EFS), response depth and disease-specific molecular milestones, toxicity profiles (Grade 3–4 adverse events and specific toxicity annotations), health-related quality of life (HRQoL), and study design framework [79].

An overview of ESMO-MCBS:H v1.0 for all major clinical trials accurately reviewed by authors (S.M. and D.A.) is shown in Table 1. Any scoring discrepancies between the assessors were systematically resolved through structured consensus discussion against ESMO-MCBS:H v1.0 evaluation forms and benchmark criteria. Next-generation targeted therapies overwhelmingly dominate the top tier. Acalabrutinib consistently earns Grade 4 status across diverse clinical settings—whether evaluated head-to-head against first-generation ibrutinib in high-risk disease (ELEVATE-RR), against chemoimmunotherapy or idelalisib–rituximab (ASCEND), or in chemo-free combination regimens with obinutuzumab or venetoclax (ELEVATE-TN, AMPLIFY). Zanubrutinib similarly achieves Grade 4 standing in frontline *TP53* wild-type disease (SEQUOIA Arm A), as does the non-covalent BTK inhibitor pirtobrutinib in heavily pretreated, post-cBTKi settings (BRUIN-CLL-321). In parallel, fixed-duration venetoclax combined with obinutuzumab reaches Grade 4 in frontline care (CLL14). By contrast, first-generation ibrutinib secures Grade 4 marks in frontline settings, either as monotherapy (RESONATE-2) or paired with venetoclax (GLOW) or rituximab (ECOG-ACRIN E1912). Evidence for intermediate benefit (Grade 3) relies predominantly on single-arm, biomarker-selected cohorts in high-risk populations, such as *TP53*-deficient or del(17p) disease (e.g., SEQUOIA Arm C, M13-982, RESONATE-17), where the absence of a randomized control arm triggers the Form 3 score cap.

Complementing clinical scales, Health Technology Assessment (HTA) bodies evaluate the economic impact and cost-effectiveness of novel cancer therapies [80]. While US health systems evaluate value primarily through total cost of care and out-of-pocket prescription drug co-pays, international HTA authorities—such as the UK’s National Institute for Health and Care Excellence (NICE) and Germany’s IQWiG—rely on strict Incremental Cost-Effectiveness Ratios (ICERs) per Quality-Adjusted Life Year (QALY) gained [81]. Under these rigid cost-effectiveness thresholds, continuous oral therapies face substantial long-term economic hurdles compared to time-limited, fixed-duration regimens. Continuous oral strategies entail open-ended, cumulative pharmaceutical expenditures that compound over years, whereas fixed-duration regimens consolidate financial outlay into a predictable treatment window, providing health systems and payers with long-term budget predictability [81].

To address the economic considerations surrounding targeted agents in CLL, it is essential to evaluate immediate acquisition costs (Table 2). This economic disparity is strongly substantiated by real-world evidence. A real-world US Medicare analysis (2016–2021; *N* ≈ 1700) demonstrated that first-line, fixed-duration VO yields superior long-term cost savings compared with continuous BTKi therapy in CLL [82]. During the initial 12 months, risk-adjusted all-cause monthly healthcare costs were comparable between VO ($13,887) and continuous BTKis ($14,492). Although VO generated higher upfront medical expenses—driven by intravenous obinutuzumab administration and protocol-mandated TLS monitoring—these initial outlays were rapidly counterbalanced by the continuous, high-volume pharmacy costs of ongoing BTKi therapy [82].

The economic trajectories diverged sharply in months 13–18 following the completion of fixed-duration VO. Upon treatment cessation, average monthly healthcare expenditure for VO patients plummeted by 67% to $4462, whereas monthly expenditures for patients on continuous BTKis remained essentially flat at $13,051 (a nominal 10% reduction). Payers and health economists assessing long-term cost-effectiveness must account for this widening cumulative cost gap, as even substantial price concessions for individual BTKis (such as ibrutinib) fail to bridge the financial divide created by indefinite treat-to-progression strategies [82].

Beyond direct drug acquisition, resource utilization and administration logistics significantly influence real-world economic evaluations. A recent Italian multicenter study assessed the organizational and economic burden of five frontline regimens for untreated CLL: fixed-duration ibrutinib plus venetoclax (I + V),VO, bendamustine plus rituximab (BR), chlorambucil plus obinutuzumab (Clb-Obi), and FCR [83]. Utilizing the product literature, clinical management protocols, and survey data across four Italian medical centers, the analysis quantified healthcare staff commitment, pharmacy drug preparation, administration time, and long-term follow-up care [84].

The administration route emerged as the principal driver of health system resource utilization. Total clinical personnel time ranged from 81.6 h (€3942) for intravenous BR to 133.7 h (€8631) for VO, which requires intensive monitoring and infusion schedules. Conversely, the all-oral fixed-duration I + V combination demonstrated a highly favorable organizational cost profile (€5724)—substantially below Ven-Obi and comparable to Clb-Obi—despite offering a fully targeted, chemo-free treatment paradigm [84].

To address the economic considerations surrounding targeted agents in CLL, it is essential to evaluate both immediate acquisition costs and long-term cost-effectiveness metrics [85].

## 9. Conclusions

The management of CLL is entering a phase in which therapeutic innovation increasingly intersects with health-system stewardship in promoting high-value care that optimizes patient QoL while minimizing treatment-related toxicity and health system costs [85,86]. As treatment efficacy has improved, the concept of therapeutic value has expanded beyond conventional measures of disease control to encompass toxicity, treatment duration, quality of life, financial expenditure, healthcare resource utilization and patient preferences [86]. The objective of therapy has therefore shifted from maximizing treatment intensity or duration toward achieving durable disease control with the most favorable overall balance between clinical benefit and treatment burden [45,87].

To establish value-based care as an integral component of CLL management, clinical trials should broaden their assessment of therapeutic benefit beyond conventional efficacy endpoints [45]. Treatment-free survival should be prospectively evaluated alongside PFS and OS, while longitudinal patient-reported outcomes should capture the differential burden of continuous versus fixed-duration therapy [45]. Economic analyses should adopt a pathway-wide perspective, incorporating treatment acquisition, monitoring, toxicity management, subsequent therapy, retreatment, and patient-level financial burden [74]. Prospective evaluation of MRD-guided treatment adaptation is also essential to determine whether response-based treatment duration can safely reduce unnecessary exposure [74,78]. Finally, trial interpretation should emphasize absolute rather than solely relative treatment effects, integrating differences in PFS, OS, treatment-free and quality-adjusted survival, and duration of treatment exposure [71].

**Figure 2:**
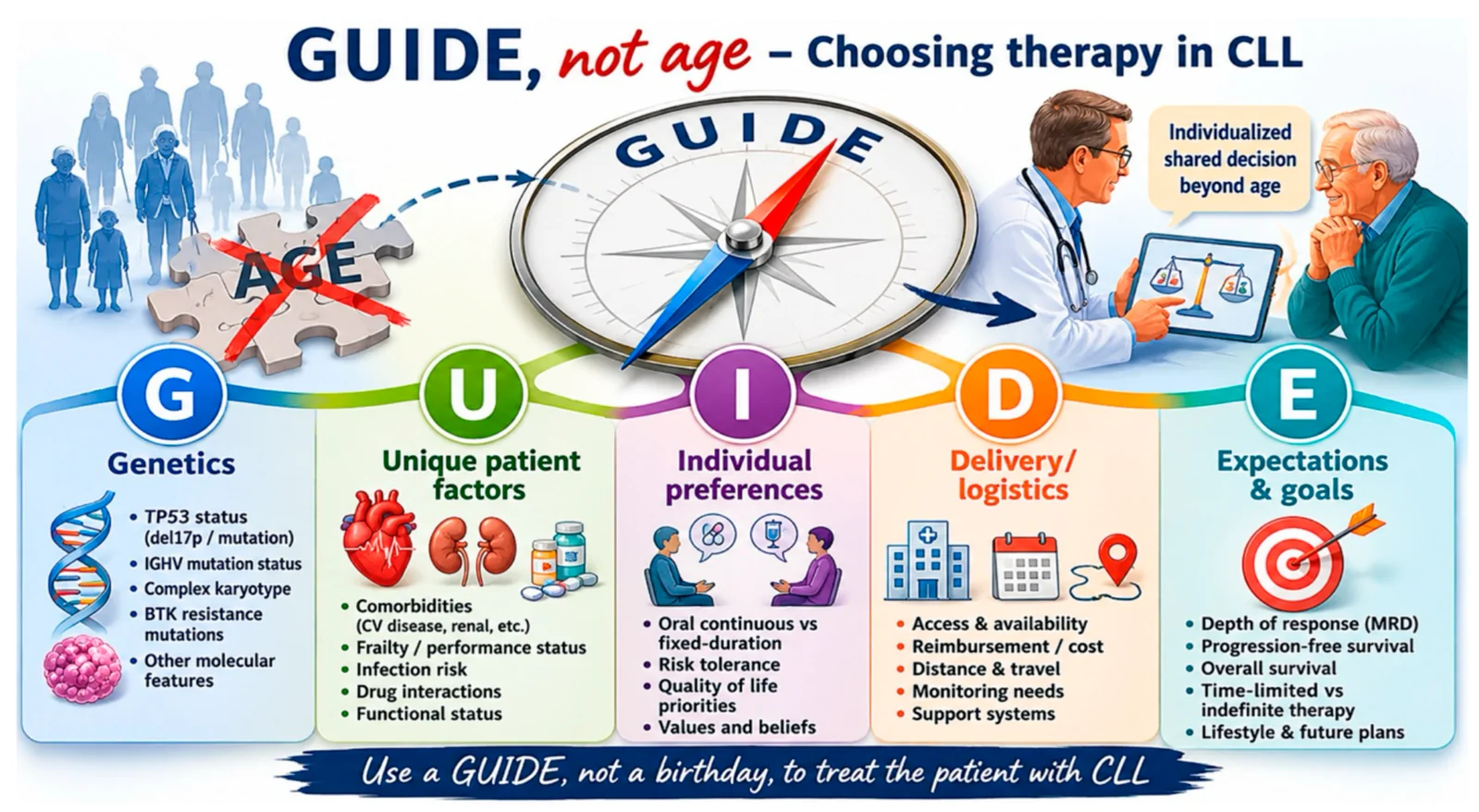
**The GUIDE Framework for Individualized Treatment Selection in Chronic Lymphocytic Leukemia (CLL).** (This figure was generated with the assistance of an AI platform).

Current clinical guidance, including the 2026 EHA CLL guidelines, increasingly reflects this broader perspective [45]. Treatment selection is moving beyond chronological age and conventional fitness assessments toward an integrated evaluation of disease biology, patient characteristics, treatment objectives, and treatment feasibility [45]. To facilitate shared decision-making in routine care, we propose the GUIDE framework—an unvalidated tool developed on the basis of the data analyzed in this review and the recent EHA guidelines—focusing on: Genetics (*TP53* and *IGHV* status), Unique host factors (comorbidities and organ function), Individual patient preferences (treatment duration vs. continuous control), Delivery logistics (access and health-system resources), and Expectations (treatment goals and survival priorities) (Figure 2).

These operational considerations highlight an important distinction between value-based care and simple cost containment. The objective is not to minimize expenditure independently of clinical outcomes, but to optimize the relationship between therapeutic benefit and the total burden generated by treatment [83]. Survival, disease control, toxicity, quality of life, treatment duration, patient preferences, and resource utilization should therefore be considered as interconnected dimensions of clinical value.

**Figure 3:**
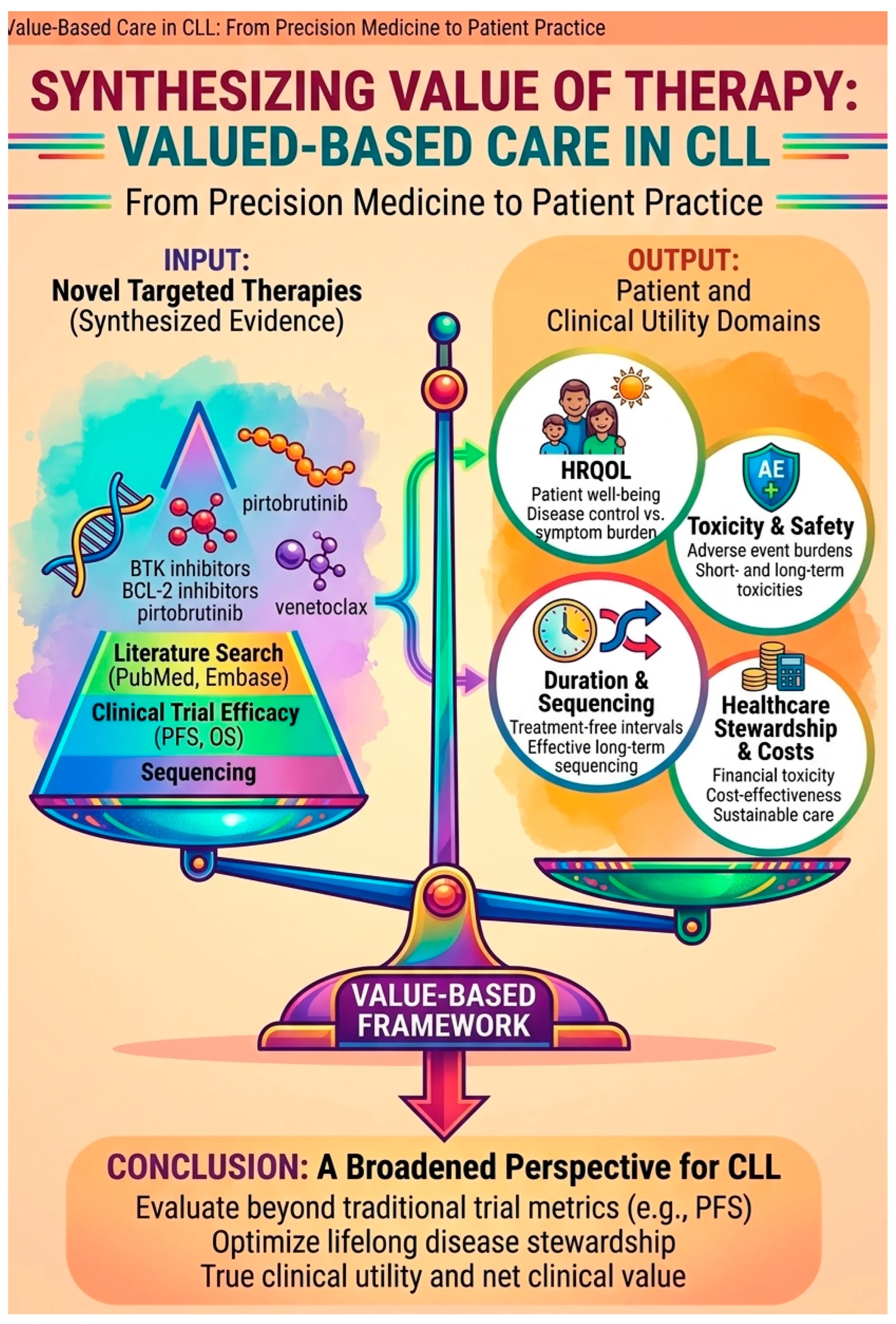
**Conceptual framework for value-based care in chronic lymphocytic leukemia (CLL).** Schematic representation illustrating the synthesis of novel targeted therapies (inputs) and patient-centered clinical utility domains (outputs) to establish a comprehensive value-based evaluation framework. This figure was generated with the assistance of an AI platform.

Finally, contemporary management of CLL is increasingly best understood not as a rigid treatment algorithm, but as a dynamic, multidirectional decision framework in which three equally important domains—biological precision, patient-centered utility, and health-system stewardship—continuously interact. Biological characteristics inform treatment selection; patient characteristics, preferences, tolerability, and quality of life shape therapeutic implementation; and economic, regulatory, and infrastructural factors influence feasibility and access (Figure 3).

Importantly, these dimensions are reciprocal rather than hierarchical, with changes in one continuously reshaping decisions across the others throughout the disease trajectory. Value-based care therefore requires an ongoing balance between disease control, patient well-being, and responsible resource utilization to optimize long-term outcomes.

Translating these principles into routine practice will also require adaptations in care delivery.

## Figures and Tables

**Table 1:** Standardized ESMO-Magnitude of Clinical Benefit Scale for Hematological Malignancies (ESMO-MCBS:H v1.0) Evaluation of Targeted Therapies Across Major CLL/SLL Clinical Trials.

Trial Name	Tested Agent(s)	Control Arm	Disease Status/17p(del) or TP53 Aberrations	Key Result	Score Adjustment/Modifiers	ESMO-MCBS:H	Rationale for Assigned Score
SEQUOIA Arm C (Phase 2)	Zanubrutinib	None	TN, 100% with del (17p)	18-mo PFS: 88.6%; ORR: 94.5%	Single-arm cap applies (Max Grade 3)	3	Non-randomized trial in high-risk genomic cohort.
SEQUOIA, Arm A (Phase 3 trial)	Zanubrutinib		TN, (wild-type *TP53*)	PFS: HR 0.42	Significant PFS extension + reduced toxicity	4	Substantial PFS reduction (HR < 0.65), favorable safety profile compared to CIT.
MURANO (Phase 3 trial)	Venetoclax + Rituximab	BR	R/R	PFS: HR 0.19	Fixed-duration therapy; sustained OS benefit	4	Improved PFS, Long-term OS improvement over BR.
M14-032 (Phase 2 trial )	Venetoclax	Single arm	R/R post-cBTKi failure	ORR: 65%; Median PFS: 24.7 months	Single-arm cap applies (Max Grade 3)	3	Single-arm study in R/R CLL; capped at Grade 3 despite high clinical utility.
CLL14 (Phase 3 trial)	Venetoclax + Obinutuzumab	Obinu + CLB	TN CLL (Comorbid)	PFS HR 0.33	Off-treatment gain + uMRD depth + QoL gains	4	PFS extension (HR < 0.65), high uMRD rates, and TFI and QoL benefits.
M13-982 (Phase 2)	Venetoclax	Single arm	R/R, 98% with del (17p)	ORR: 79.4%; 24-mo PFS: 54%	Single-arm cap applies (Max Grade 3)	3	Single-arm registration trial in high-risk del (17p); capped at Grade 3.
ASCEND (Phase 3 trial)	Acalabrutinib	Investigator’s choice Idela + R or BR	R/R CLL	PFS: HR 0.31	Favorable safety profile vs. IdR	4	Meaningful PFS superiority with acalabrutinib and reduced toxicity.
ELEVATE-TN (Phase 3 trial)	Acalabrutinib + Obinutuzumab	Obinu + CLB	TN CLL	PFS HR 0.10	Sustained 6-year PFS advantage + safety	3	Reduction in risk of progression/death (HR < 0.65) vs. acalabrutinib. Increased toxicity (infections).
ELEVATE-TN (Phase 3 trial)	Acalabrutinib	Obinu + CLB	TN CLL	PFS HR 0.21	Sustained 6-year PFS advantage	4	Single-agent acalabrutinib PFS improvement with (HR ≤ 0.65). Reduced cardiovascular toxicity.
ELEVATE-RR (Phase 3 trial)	Acalabrutinib	Ibrutinib	R/R; High-risk (17p-del, 11q-del)	Non-inferior PFS (HR 1.00); AF risk 9.4% vs. 16.0%	Safety improvement (reduced cardiovascular toxicity)	4	Non-inferior efficacy with statistically significant reduction in Grade 3+ cardiac adverse events and discontinuations.
AMPLIFY (Phase 3 trial)	Acalabrutinib + Venetoclax + Obinu	FCR or BR	TN CLL; younger/fit (Wild-type *TP53*)	PFS HR 0.27	Fixed-duration triplet efficacy	3	Significant PFS improvement (HR < 0.65). Increased toxicity (infections especially COVID19).
AMPLIFY (Phase 3 trial)	Acalabrutinib +Venetoclax	FCR or BR	TN CLL; younger/fit (Wild-type *TP53*)	PFS HR 0.42	Fixed-duration doublet efficacy	4	All-oral doublet achieves PFS HR < 0.65 over CIT.
BRUIN (Phase 2)	Pirtobrutinib	Single arm	R/R; previous cBTKi	ORR: 74%; Median PFS: 19.4 months	Single-arm cap applies (Max Grade 3)	3	Effective non-covalent BTKi in heavily pretreated cohort; capped at Grade 3 per Form 3 rules.
BRUIN CLL-321 (Phase 3 trial)	Pirtobrutinib	Investigator’s choice (Idela + R or BR)	R/R; previous exposed cBTKi	PFS HR 0.44	Target population with post-cBTKi; unmet need	4	Superior PFS (HR < 0.65) in post-cBTKi disease. Excellent safety profile.
RESONATE (Phase 3 trial)	Ibrutinib	Ofatumumab	Relapsed/Refractory CLL	PFS HR 0.22; OS HR 0.43	Significant PFS and OS gains	4	Demonstrated major PFS (HR < 65) and OS benefit over monotherapy control arm.
RESONATE-2 (Phase 3 trial)	Ibrutinib	Chlorambucil	TN CLL (Wild-type *TP53*)	PFS HR 0.16; OS HR 0.16	Sustained 10-year PFS/OS benefit	4	Frontline monotherapy yielding profound PFS and OS hazard ratio reductions (HR < 0.65).
GLOW (Phase 3 trial)	Ibrutinib +venetoclax	Chlorambucil + obinu	TN CLL (Wild-type *TP53*)	PFS HR 0.21	Fixed-duration therapy, deep uMRD gains	4	All-oral fixed-duration doublet achieving superior PFS (HR < 0.65) and deep residual disease clearance.
CLL12 (Phase 3 trial)	Ibrutinib	Placebo	Early stage/Asymptomatic high-risk CLL	Event-Free Survival (EFS) HR 0.25	No demonstrated OS benefit	2	Demonstrated EFS extension in early-stage watch-and-wait CLL without OS improvement; modest score per Form 1 criteria.
ECOG-ACRIN E1912 (Phase 3 trial)	Ibrutinib +R	FCR	TN CLL (Age ≤ 70, (17p)del excluded)	PFS HR 0.35	Long-term OS gain in younger fit cohort	4	Statistically significant improvement in both PFS and OS (HR < 0.65) compared to CIT.
ILLUMINATE (Phase 3 trial)	Ibrutinib + Ofatumumab	Chlorambucil + obinu	TN CLL	PFS HR 0.23	Significant PFS extension	3	PFS HR < 0.65 meeting criteria for Grade 4 benefit in frontline unfit setting.
RESONATE-17 (Phase 2 trial)	Ibrutinib	Single arm	R/R CLL with (17p)del	ORR: 83%; 24-mo PFS: 63%	Single-arm cap applies (Max Grade 3)	3	Pivotal single-arm cohort in high-risk genomic disease; capped at Grade 3 per Form 3 criteria.
ALPINE (Phase 3 trial)	Zanubrutinib	Ibrutinib	R/R CLL	36 mo PFS 54.4%	Safety improvement (reduced cardiovascular toxicity)	3	HR 0.68; QoL not quantified.

Abbreviations: ORR, Overall Response Rate; PFS, Progression-free Survival; TN, Treatment-naïve; BR, Bendamustine–Rituximab; HR, Hazard Ratio; CIT, Chemo-immunotherapy; R/R, Relapsed/Refractory; OS, Overall Survival; CLB, Chlorambucil; QoL, Quality of Life; Idela-R, Idelalisib–Rituximab; FCR, Fludarabine, Cyclophosphamide, Rituximab; TFI, Treatment-free interval; cBTKi, covalent Bruton tyrosine kinase inhibitor.

**Table 2:** Approximate Economic Profile and Cost-Effectiveness of Key Targeted Treatment Regimens in CLL.

Treatment Regimen	Regimen Type & Duration	Primary Cost Drivers	Approximate Cost Profile (US Payer Baseline) *	Cost-Effectiveness/Value Considerations
Ibrutinib (Continuous)	Oral BTKi; daily until progression	High cumulative drug acquisition; AE management (atrial fibrillation, hypertension, bleeding)	~$130,000–$150,000/year (continuous)	Higher overall lifetime cost; less cost-effective relative to 2nd-gen BTKis due to toxicity management burden.
Acalabrutinib ± Obinutuzumab	Oral 2nd-gen BTKi; daily until progression	Drug acquisition; infusion & TLS monitoring costs if combined with obinutuzumab	~$135,000–$155,000/year (continuous)	Cost-effective vs. ibrutinib due to lower rate of Grade ≥ 3 cardiovascular toxicity and reduced medical resource utilization.
Zanubrutinib (Continuous)	Oral 2nd-gen BTKi; daily until progression	Drug acquisition; routine lab and clinical monitoring	~$135,000–$150,000/year (continuous)	Favorable incremental cost-effectiveness ratio (ICER) vs. ibrutinib; highly cost-effective option in both treatment-naïve and R/R CLL.
Venetoclax + Obinutuzumab (VenO)	Fixed-duration; 12 months (12 cycles)	Upfront venetoclax ramp-up, TLS monitoring/prophylaxis, obinutuzumab infusions	~$140,000–$165,000 (total 1-year course)	High Long-Term Value: High initial expense offset by 0 drug costs during off-treatment periods (TFS), leading to lower 5- and 10-year total costs.
Ibrutinib + Venetoclax (IV)	Fixed-duration; 15 cycles (CAPTIVATE profile)	Dual targeted drug acquisition during active treatment period	~$180,000–$210,000 (total 15-month course)	Fixed treatment boundary prevents indefinite spending; cost-effective in non-high-risk profiles yielding deep uMRD and extended PFS.
Chemoimmunotherapy (FCR/BR)	Fixed-duration; 6 cycles	Inpatient/outpatient management of severe neutropenia, infection, and secondary malignancies	~$15,000–$35,000 (total course)	Low drug acquisition costs; however, lower long-term QALY gains and higher toxicity-related hospitalization costs reduce overall cost-effectiveness relative to targeted agents in unfit or high-risk patients.

* Note: Figures represent estimated wholesale acquisition/annual healthcare costs based on published US health-economic models and real-world cost comparisons. Exact expenditures vary depending on regional health system pricing, discounts, and toxicity management.

## Data Availability

Data available on request from the authors.

## Supplementary Materials

The following supporting information can be downloaded at: https://www.mdpi.com/article/10.3390/cancers18213462/s1, Supplementary Table S1: Preferred Reporting Items for Systematic reviews and Meta-Analyses extension for Scoping Reviews (PRISMA-ScR) Checklist; Details on PRISMA Complete Search Strategies.
